# Children of the Revolution: The Impact of 1960s and 1970s Cultural Identification on Baby Boomers’ Views on Retirement

**DOI:** 10.1177/01640275211068456

**Published:** 2022-03-25

**Authors:** Orlaith Tunney, Kène Henkens, Hanna van Solinge

**Affiliations:** 1 Netherlands Interdisciplinary Demographic Institute (NIDI-KNAW), The Hague, the Netherlands; 2 University Medical Center Groningen, Groningen, the Netherlands; 3 Department of Sociology, University of Amsterdam, Amsterdam, the Netherlands

**Keywords:** retirement, retirement patterns, identity, ageing, older worker

## Abstract

There is widespread speculation that baby boomers will make significant changes to the retirement landscape. Some attribute these changes, at least in part, to countercultural movements this generation pioneered during the sixties and seventies. However, empirical investigation into the long-term impact of countercultural identification in youth is scarce. To address this, our study examines associations between baby boomers’ retirement views and identification with counterculture. Using data from 6024 pre-retired Dutch older workers, we investigate whether greater identification with counterculture is associated with more active retirement views. Our results show that greater identification with counterculture is associated with more active retirement views, even when controlling for potential confounders. Beyond highlighting the diversity of the baby boom generation, these findings support the idea that (counter)cultural identity in youth has an impact across the life course and may therefore have implications for other key questions of life’s third age beyond retirement.

## Introduction

The nineteen sixties and seventies witnessed a revolution in attitudes and cultural norms in terms of music, sexuality, drugs and politics (Braunstein & Doyle, 2002). These changes were driven by countercultural movements – the hippy movement, anti-war movements, civil rights movements, second wave feminism and the gay rights movement, amongst others (Chalmers, 2012). Those who came of age during this revolution, so-called baby boomers, are credited with breaking the mould of the traditional life course (Gilleard & Higgs, 2002) and radically changing societal norms including marriage and living arrangements that are associated with the second demographic transition (Van de Kaa, 1987). Consequently, there has been widespread speculation about the legacy of this generation (Martin & Roberts, 2021, and whether they will also inspire wholesale changes to the next milestone they encounter, retirement (Harkin & Huber, 2004).

Accounting for the cultural climate in which they grew up may help us understand how and why baby boomers may retire differently than previous cohorts (Gilleard & Higgs, 2002). Hamilton and Hamilton (2006) posit that maturing during the unique period of cultural change of the sixties and seventies is responsible, at least in part, for the reformulation of retirement we see and expect amongst baby boomers. This suggestion fits with the notion that identification with youth culture can be highly influential in shaping identity and subsequent experiences (Kehily, 2007). However, others have called into question the very idea that baby boomers will reinvent retirement, positing instead that baby boomers are merely continuing changes to retirement instigated by their parents’ generation (Chambré & Netting, 2018). Moreover, despite its intuitive appeal, links between sixties and seventies counterculture and perceptions of retirement remain unexplored. To address this, our study will investigate whether identification with countercultures of the sixties and seventies are linked to the retirement views found amongst baby boomers.

Our study focuses on the retirement views of near-retirement older workers. Understanding these individuals’ perceptions of future events such as retirement is vital, as mental representations people form of who they could or should become may influence the way they organize and evaluate their actual and future development (Frazier et al., 2002). Differently put, how people picture retirement may shape motivation and behaviour in the present. Better understanding how individuals perceive and experience retirement may be beneficial not only in terms of predicting retirement behaviour and outcomes, but in guiding practitioners and policy makers seeking to help those navigating the retirement transition (Maggiori et al., 2014).

Various retirement typologies have been developed to explore and better understand perceptions and experiences of retirement. The most widely known are those of Hornstein and Wapner (1986) and Schlossberg (2004). Hornstein and Wapner (1986) identified four main retirement styles: Transition to old age/rest: where retirement involves slowing down and diminishing activity; New beginning: a new phase of life where retirees focus on their own needs and goals rather than those of others; Continuation: where retirement gives freedom to pursue existing interests and activities – including work – in a more relaxed, self-directed way; and Imposed disruption: where retirement is the loss of a highly valued activity. Schlossberg (2004) identifies five distinct retirement types. The Continuer and Adventurer styles match closely with the Continuation and New Beginning styles of Hornstein and Wapner (1986). The remaining styles are: Easy gliders who value the freedom offered by retirement; Searchers who are uncertain and indecisive around retirement; and Retreaters who tend to disengage from life entirely in retirement.

While these qualitative investigations of retirement views have moved the literature beyond an outdated one-size-fits-all view of retirement, there remains a dearth of quantitative literature surrounding these retirement typologies. Despite recent attempts to develop and validate empirical measures of retirement styles (e.g. Maggiori et al., 2014), little is known about the prevalence and distribution of various retirement views within the general population, nor factors associated with these views.

Our study aims to address the paucity of empirical investigations into both retirement views and the long-term impact of identification with counterculture A large sample (*N* = 6024) of pre-retired Dutch older workers aged between 60 and 65 at the time of data collection (2015) were recruited for the current analysis. This study will be the first to empirically test the assumption of Hamilton and Hamilton (2006) – that the experience of maturing in the 1960s and 1970s may shape the retirement views of the baby boom generation. In doing so, we will make several contributions to the literature. First, to the best of our knowledge, this study provides one of the few large-scale, prospective, empirical investigations of the retirement views of working baby boomers. Second, we take the novel approach of measuring identification with cultural movements of their youth, such as the hippy movement, feminism and anti-establishment. In doing so, we can investigate the impact of countercultural identity on retirement views. Approaching this generations’ retirement views from a cultural rather than a more traditional cohort perspective, offers a more nuanced and broader understanding of their impact on and relationship to retirement (Gilleard & Higgs, 2007). Additionally, this approach allows us to examine within-cohort differences along lines of cultural identification, an important strength given the tendency in previous literature to treat this diverse cohort as a homogenous group (Hughes & O’Rand, 2004).

## Conceptual Framework

### Generation and Identity

The notion that we garner a sense of identity from our generation is pervasive in modern society (Willetts, 2010). Generations are usually viewed as birth cohorts, comprising a set of common experiences and historical and geographical locations (Kertzer, 1983). Alternatively, they may be viewed through a more cultural lens, with generations described as cultural constructs involving historical participation guided by individuals’ consciousness (Mannheim, 1970). The latter approach, in which the idea of social generations is explored, has garnered increased research attention in recent years (Roberts & France, 2021). While some have argued for the superiority of a cultural approach over a cohort approach to understanding the retirement of the baby boom generation (Gilleard & Higgs, 2007), there appears at least to be consensus that shared experiences are crucial in shaping generations and generational identity. Those in our study shared the experience of coming of age during the 1960s and 70s, a period of profound social and cultural change. It is this experience of the post-war cultural revolution that is believed to have moulded the baby boomer generation and established their generational consciousness (Gilleard & Higgs, 2002). However, while members of this cohort all witnessed these societal changes, the extent to which they identified with and participated in countercultural movements driving these changes may vary widely (Weisner & Bernheimer, 1998). As such, the effects these countercultural identities can be expected to exert on individuals throughout the life course may differ substantially.

### The Importance of Youth in Identity Formation

The view that the form identity takes during adolescence significantly impacts later life is common among social scientists (Kinney, 1993), and the belief that generational identity is formed in youth is why links between social change and generations is most commonly investigated through youth (Roberts & France, 2021). The complexity of the concept of identity makes empirical research on the subject challenging (Stryker & Burke, 2000). However, identity is generally believed to emerge and solidify in adolescence (Klimstra & van Doeselaar, 2017). Support for the notion that youth is central in identity development can also be inferred from cognitive neuroscientific research, which finds that functional and structural changes in the brain between the ages of 10 and 20 may reflect a sensitive period for adaption to an individuals’ social environment (Blakemore & Mills, 2014). That said, meta-analytic evidence indicates that identity is by no means solidified by adolescence or early adulthood, and identity development generally strengthens with age (Kroger et al., 2010). This is echoed in calls to extend the period we view as crucial in the life course to include much of the twenties, or ‘emerging adulthood’ (Wood et al., 2018).

### Youth Cultural Identity and the Life Course

A common assumption in popular culture, found also in academic literature, is that underpinning changes made by baby boomers to the life course thus far is a desire to remain youthful. The countercultural movements they witnessed or engaged in were cultures of youth. So, when faced with what these countercultures opposed – the trappings of age – this generation preferred to ignore or redefine what it meant to get older (Gilleard & Higgs, 2007). Many believe this pattern will continue as boomers reach the third age, with some commentators predicting that their desire to avoid traditional notions of old age will usher in an era of more active retirement in terms of lifestyle, consumption and participation in the labour force (Harkin & Huber, 2004). Others more directly link this reformulation of retirement to attachment to youth culture stemming from the countercultural movements of the sixties and seventies (Hamilton & Hamilton, 2006). However, the notion that baby boomers will truly reinvent the retirement landscape, or are the sole arbiters of these changes, is not universally accepted. Others believe changes they may bring to retirement are more accurately viewed as a continuation of a long-term shift in retirement initiated by the preceding generation (Chambré & Netting, 2018).

Indeed, while links between the cultural aspects of their youth to baby boomers’ retirement hold intuitive appeal, empirical investigations on the impact of youth identity later in the life course remain scarce. Weisner and Bernheimer (1998) followed 254 US-based families from the mother’s third trimester of pregnancy (1974–1975) to when the children were in their late teens (1993–1994). Through a series of interviews and home observations, they investigated the impact of countercultural identities in youth on parenting and beliefs in midlife. They found identification with 1960s counterculture did have some impact on parenting, child outcomes and parents’ beliefs – with consistently high identification with counterculture over time in the parents even associated with a protective effect against substance abuse problems and school related issues amongst their offspring.

Another study by D. E. Sherkat (1998) investigated the impact of countercultural (protest) participation in the 60s and 70s – along with life-course factors, and traditional agents of socialization – on religious beliefs and practices. They found that countercultural participation had a significant negative effect on participants’ (*N* = 1034) religious beliefs over the 17-year study period (1965–1973). While life-course factors and agents of socialization were found to have relatively stronger effects on religious orientation, the results nevertheless indicate the long-term impact of countercultural identification. Therefore, while these works, and subsequent follow up studies (Weisner, 2001), did not investigate the impact of youth countercultural identity on retirement specifically, they nevertheless lend credence to the idea that countercultural identities developed in the 1960s and 1970s may shape the lives of baby boomers, in later life.

In marketing and consumer psychology research, evidence has been found indicating that an individual’s past identity can impact their pattern of behaviour during retirement. Through a series of semi-structured interviews with US retirees (*N* = 65), Schau et al. (2009) investigated identity-related consumption patterns amongst retirees. They found identity-related activities and projects were put on hold after adolescence, but often re-emerged in retirement. This ‘identity renaissance’ was often cited by participants as shaping their behaviour and consumption in old age. However, while youth identities played a role in the consumption pattern of some retirees, this effect was not universal, with new consumption patterns and identities also emerging alongside or instead of the revival of identities from youth.

Recent studies have also highlighted the importance of the baby boom generations’ values in influencing behaviours in later life such as the provision of childcare for grandchildren (Airey et al., 2021). However, links between these values and (counter)cultural experiences in youth were not explicitly made, nor was retirement or views surrounding it a central focus of the work. Therefore, while these studies can be seen as offering preliminary support for the idea that past experiences, identities and values are important in retirement, they also highlight the need for additional investigation into whether, and to what extent, countercultural identification impacts the retirement of members of the baby boom generation.

### Countercultures of the 1960s and 1970s

This lack of empirical investigation into the long-term impact of identification with countercultures of this era is hardly surprising given the amorphous nature of the countercultural movements themselves. While the hippy movement may loom largest in public consciousness, perhaps owing to their distinct aesthetic (Moretta, 2017), this movement alone does not define the era. Protest culture – from civil rights marches to anti-war and environmental protests (D. S. Sherkat & Blocker, 1993) – was a key feature of the age. The label anti-establishment also emerged in the sixties to describe a variety of groups opposed to the prevailing societal institutions and values. This era also witnessed a growing drug culture – with rapid increases in legal and illegal substance use across Western societies (Marchant, 2013); and the proliferation in alternative lifestyles such as non-marital cohabitation and homosexual relationships. (Rubin, 2001). Calls for women’s rights grew louder too, with the rise of second wave feminism and the women’s liberation movement (Freeman, 1973). The sixties (Marwick, 2011), and to a larger extent seventies, are often associated with increased individualism. So much so that the latter has been dubbed the ‘Me Decade’ in some quarters (Wolfe, 1976). Thus, rather than a unified entity, the counterculture of the time was made up of varied subcultures that permeated and transformed society (Marwick, 2011).

Our study will investigate the association between identification with these countercultural movements and the retirement views of near-retirement baby boomers (born 1950–1955) in the Netherlands. Given previous assertions that members of this generation wish to maintain their youthful identity and eschew the idea of growing old and inactive (Harkin & Huber, 2004), we hypothesize that greater identification with counterculture will be linked to more ‘active ’retirement views such as the New beginning and Continuer retirement views (hypothesis 1a). In contrast, we expect less identification with counterculture to be associated with the more ‘inactive’ Freedom From Work retirement view, and the more avoidant Searcher and Retreater retirement view (hypothesis 1b).

### Design and Method

#### Data

Data in the current study was taken from the first wave of the NIDI Pension Panel Study (NPPS), a prospective cohort study conducted in 2015 (Henkens et al., 2017). Participants were drawn from the three largest Occupational Pension Funds in the Netherlands. A stratified sampling procedure based on organizational size and sector was used. Within this stratified sample, participants were randomly drawn from those who were aged 60–65 and worked at least 12 hours per week (*N* = 15,480). Participants received a hardcopy of the questionnaire from their pension fund provider but could also choose to complete the questionnaire online. A reminder letter was sent to participants 2 weeks following the start of data collection, with another reminder sent 6 weeks later to those who still had not completed the questionnaire. A total of 6793 completed questionnaires were returned, giving a response rate of 44 percent. Participants who received a shortened version of the questionnaire with some key independent variables omitted (*n* = 499), and those who failed to respond to the dependent variable (*n* = 270), were excluded from the sample. This left a total of 6024 participants for further analysis. Item non-response was generally low (average of 3.09%), ranging from 0 to a maximum of 7.98% for our measure of wealth. Given the relatively high percentage of missing data in some variables we will use multiple imputation by chained equations (MICE) in which 20 imputed datasets are generated. Estimates reported will be obtained using the *mi estimate* command (Stata Version 16) to control for variability between imputations when reporting coefficients and standard errors.

### Measures

#### Dependent variables

Building on the retirement styles outlined in the work of Hornstein and Wapner (1986) and Schlossberg (2004) we developed a brief measure of identification with retirement views, the Short Measure of Retirement Views. Participants were asked to choose which of the following descriptions of retirement suited them best: (a) ‘Retirement means enjoying the fact that you are no longer working’; (b) ‘Retirement is something I’d rather not think about’; (c) ‘Retirement means that you finally have time to develop yourself and learn new things’; (d) ‘Retirement means continuing work activities, but at a slower pace’ or (e) ‘Retirement is still unknown ground for me’. Participants’ selection on this variable was taken as indicative of their preferred retirement view. These views were subsequently labelled Freedom From Work, Retreater, New Beginning, Continuer and Searcher.

#### Independent variable

The primary independent variable in our analysis is identification with counterculture. Participants were asked ‘To what extent did you identify with the characteristics of the 1960/70s in your youth’. Respondents then indicated on a scale from 1 (a lot) to 4 (not at all) how much they identified in their youth with (1). Hippy culture, (2). Protest culture, (3). Individualism, (4). Feminism, (5). Drugs culture, (6). Anti-establishment culture and (7). Alternative lifestyles. These items were reverse coded so that a higher number indicated a greater identification with this aspect of counterculture. These items were then combined to form a countercultural identity scale, which showed good reliability and internal consistency (α = .81). Factor analysis further confirmed these items best followed a unidimensional structure.

#### Control variables

Several demographic and individual characteristics of participants were included as control variables. Participants were asked to state their gender (Male or Female), and their partner status (Living alone or living with partner). Participants were then asked to indicate the highest level of education they attained from a list ranging from 1 (primary school) to 7 (university degree). Education levels were based on the International Standard Classification of Education (ISCED). Based on previous work (Idler & Benyamini, 1997), subjective health was measured by asking participants ‘How would you characterize your health in general?’ Answers were given on a scale of 1 (excellent) to 5 (very poor). This scale was then reverse coded so that a higher score indicated higher subjective ratings of health. Wealth was assessed by asking participants to estimate how large their total wealth (including own house, savings and stocks minus debts/mortgage) from categories ranging from 1 (less than €5000) to 7 (more than €500,000). Wealth was subsequently categorized into low (1, 2, 3), moderate (4, 5) ine and high (6, 7) levels of wealth. Participants were also asked to rate how stressful, and how physically demanding their current jobs were. Ratings for both items were given on a scale from 1 (very) to 4 (not at all). These measures were subsequently reverse coded for ease of interpretation. These measures were adopted from the Study on Transitions in Employment, Ability and Motivation (van Vegchel et al., 2004).

Additionally, we measured participants’ retirement self-efficacy using the following questions: ‘I can handle whatever comes my way in retirement’ and ‘I will definitely realize the plans I make for retirement’. Participants responded on a five-point scale ranging from 1 (completely agree) to 5 (completely disagree). These two items were then reverse coded so that a higher score indicated greater retirement self-efficacy and combined to form a retirement self-efficacy scale (α = .53, *r* = .37). Finally, we measured participants’ future time perspective using items primarily drawn from earlier work by Jacobs-Lawson and Hershey (2005) by asking participants to rate on a scale of 1 (completely agree) to 5 (completely disagree) how much they agreed with the following statements: ‘It is important to take a long term perspective’, ‘I enjoy thinking about how I will live years from now in the future’ and ‘I pretty much live on a day-to-day basis’. We then reverse-coded the first two times so that higher scores on each of the items would indicate a greater future orientation. The three items were combined to form a single future-time-perspective scale (α = .60) that was treated as a continuous measure.

The means, standard deviations and correlations for all the independent and control variables included in our analyses can be found in Table 1. The results of this correlation matrix indicate that our dependent variable, identification with counterculture, is associated with education. Better educated individuals have a stronger identification with counterculture than those with less education.

**Table 1.** Correlation Matrix of all Independent and Control Variables Included in the Analysis.

Variable	1	2	3	4	5	6	7	8	9	10
1. Counterculture	—									
2. Sex (male = 1)	.04**	—								
3. Education level	.29***	.11***	—							
4. Partner status (partner = 1)	.07***	.25***	.02	—						
5. Health	-.00	.02	.09***	−.05***	—					
6. Wealth	-.00	−.09***	.20***	−.23***	.15***	—				
7. Job stress	.08***	.01	.14***	−.02	−.13***	−.01	—			
8. Job physicality	−.12***	.03	−.40***	.01	−.17***	−.13***	.18***	—		
9. Self-Efficacy	.05***	.00	.01	−.01	.18***	.06***	−.01	.01	—	
10. Future time perspective	.03*	−.03*	.11***	−.12***	.10***	.13***	.11***	−.04**	.36***	—
Mean	1.52	.45	4.61	1.21	3.20	1.89	2.67	1.96	3.92	3.63
SD	.53	.50	1.80	.40	.87	.74	.88	1.03	.62	.68
No. of obs	5602	6024	5978	5946	5952	5543	5863	5745	5912	5885

*p ≤ .05; ** *p* ≤ .01; *** *p* ≤ .001.

*Note.* Table is based on original, non-imputed data. Due to missing data cases per variable may differ.

### Analysis

Following the exploration of descriptive statistics, Multinomial logistic regression analysis (MNL) (Stata Version 16: mlogit) was used to investigate the impact of counterculture on retirement views. MNL was selected as the nominal categorical nature of our dependent variable makes analyses using other traditional statistical methods, such as multiple regression, inappropriate for use (Zickar & Gibby, 2003). The dependent variable in the current analysis – retirement view – has five categories: ‘Freedom From Work’, ‘Retreater’, ‘New Beginning’, ‘Continuer’ and ‘Searcher.’ Contrasts of predictive effects on the dependent variable were created comparing four of the categories in the MNL analysis to the baseline category ‘Freedom From Work’. Thus, the impact of counterculture was tested for (i) ‘Retreater’ against ‘Freedom From Work’, (ii) ‘New Beginning’ against ‘Freedom From Work’, (iii) ‘Continuer’ against ‘Freedom From Work’ and (iv) ‘Searcher’ against ‘Freedom From Work’. Freedom from work was chosen as the baseline category given that it represents the most traditional notion of retirement and was the largest category. We estimated two MNL models. The first (Model 1) estimated the impact of our key independent variable countercultural identity on identification with retirement views, without controlling for possible confounding factors. The second MNL model (Model 2) investigated the association between countercultural identity and retirement view, this time with the inclusion of control variables. Clustered standard errors (Stata version 16: vce (cluster)) were used to control for the nesting of participants within organizations. All analyses were conducted using Stata version 16.1.

## Results

### Descriptive Results

As evident from Table 2, respondents in our study differ widely in their views on retirement. Of the five retirement views investigated; the majority of participants (52.5%) viewed retirement as an opportunity to rest and escape working life. Following this, one in five participants viewed retirement as a new beginning (21.5%). Less prevalent were the views of retirement as a continuation of work activities at a slower pace (12.9%), and as unknown ground (9.6%). Participants were least likely to identify with the ‘retreater’ retirement view (3.6%), in which individuals do not wish to think about retirement. As a sensitivity analysis, we also performed analyses in which participants could rate the extent to which they identified with each view rather than select only one retirement view. The results of these analyses are shown in Appendix A.

**Table 2.** Breakdown of Identification with Retirement Views amongst the Sample Population.

	*n*	%
Freedom From Work	3162	52.49
Retreater	217	3.60
New beginning	1294	21.48
Continuer	774	12.85
Searcher	577	9.58

**Figure 1.**
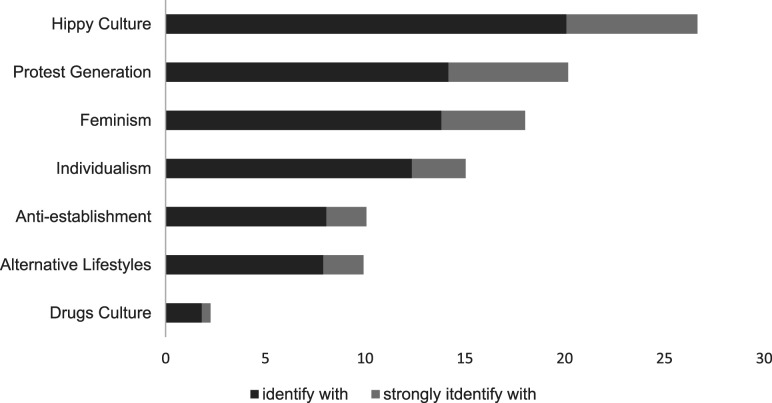
Identification with countercultural identities. *Note.* Figure is based on original, non-imputed data. Due to missing data cases per variable may differ.

Though respondents in our survey all belong to a similar cohort, their identification with counterculture in youth differs widely. Figure 1 outlines the extent to which participants identified in their youth with each of the elements of our measure of countercultural identification. Identification with the hippy culture was most prevalent, with 26.7% identifying or strongly identifying with this aspect of counterculture. Identification with the remaining countercultural movements of the 60s and 70s was as follows: Protest Generation (20.2%), Feminism (18.0%), Individualism (15.0%), Anti-establishment (10.1%), Alternative Lifestyles (9.9%) and Drugs Culture (2.3%).

### Impact of Counterculture on Retirement View

Results of the multinomial logistic regression analyses investigating the association between countercultural identity and retirement view are presented in Table 3. In these analyses, the likelihood of identification with the four retirement view categories – New Beginning, Retreater, Continuer and Searcher – is calculated relative to the base category Freedom From Work. Model 1 outlines the association between identification with counterculture retirement view without the inclusion of control variables. In keeping with hypothesis 1a, we found a significant positive association between identification with counterculture and more active retirement view such as New Beginning (*β* = .54, SE = .06, *p* < .001) and Continuer (*β* = .39, SE = .07, *p* < .001), compared to those who viewed retirement as Freedom From Work. Contrary to hypothesis 1b, a significant positive association was also found between counterculture and the Searcher (*β* = .20, SE = .08, *p* < .001) retirement view; with no significant association between counterculture and the Retreater retirement view found (*β* = −.13, SE = .16, *p* = .380).

**Table 3.** Multinomial Logistic Regression Analyses Predicting Retirement Views from Identification with Counterculture Without (Model 1) and with (Model 2) the Inclusion of Control Variables.

Variable	Outcome: Retreater *Coef. (SE)*	Outcome: New Beginning *Coef. (SE)*	Outcome: Continuer *Coef. (SE)*	Outcome: Searcher *Coef. (SE)*
Model 1: Model with key predictor variable				
Counterculture	−0.06 (.17)	0.60 (.06)***	0.40 (.08)***	0.23 (.09)**
Model 2: Model with controls				
*Key Predictor Variable*				
Counterculture	−0.11 (.18)	0.41 (.07)***	0.29 (.08)***	0.23 (.09)*
*Control Variables*				
Sex (ref. male)				
Female	0.67 (.15)***	0.23 (.07)***	0.04 (.09)	0.24 (.10)*
Education level	0.04 (.05)	0.19 (.02)***	0.15 (.03)***	0.05 (.03)
Partner status (ref. living with partner)				
Living alone	0.27 (.18)	0.32 (.09)***	0.22 (.11)	0.34 (.12)**
Health	0.14 (.09)	0.07 (.04)	0.29 (.05)***	0.19 (.06)***
Wealth (ref. low)				
Moderate	−0.54 (.19)**	−0.04 (.08)	0.01 (.10)	0.03 (.11)
High	0.30 (.20)	0.12 (.11)	0.29 (.12)*	0.21 (.15)
Job stress	−0.14 (.08)	0.02 (.04)	−0.20 (.05)***	−0.16 (.06)**
Job physicality	−0.29 (.09)***	−0.06 (.04)	−0.04 (.05)	−0.08 (.05)
Retirement self-efficacy	−0.67 (.13)***	−0.09 (.06)	−0.32 (.07)***	−0.65 (.08)***
Future time perspective	−0.29 (.11)**	0.24 (.06)***	−0.12 (.06)*	−0.46 (.08)***

*p ≤ .05; ** *p* ≤ .01; *** *p* ≤ .001.

*Note.* Base outcome = Freedom From Work. No. of observations for each analysis is 6023 when controlling for clusters within the data.

*Description of Retirement Views:* Freedom From Work = Retirement as a time to enjoy that you are no longer working; Retreater = Retirement as something you would rather not think about; New Beginning = Retirement as a time to develop yourself and learn new things; Continuer = Retirement as a continuation of work activities but at a slower pace; Searcher = Retirement is still unknown ground for me.

### Controlling for confounders

Model 2 (Table 3) shows the results of the second MNL analysis investigating the association between counterculture and retirement view with the inclusion of control variables. As with Model 1, the likelihood of identification with the four retirement view categories in this analysis is calculated relative to the base category Freedom From Work. With respect to control variables, women were more likely to identify with the Retreater, New beginning and Searcher retirement views than men. Compared to less educated individuals, better educated individuals were significantly more likely to identify with the New Beginning and Continuer retirement views. Regarding partner status, those living alone were more likely to select New Beginning and Searcher retirement views than those cohabiting with a partner. Higher subjective health was associated with greater likelihood of the Continuer and the Searcher retirement view. Moderate levels of wealth reduced the likelihood of identifying with the Retreater retirement view compared to those with lower levels of wealth, while the likelihood of selecting the Continuer view increased amongst the highest earners. Greater job stress was negatively associated with the Continuer or Searcher retirement view. Those who reported greater job physicality were less likely to fall under the Retreater view. Higher retirement self-efficacy was negatively associated with the Retreater, Continuer and Searcher styles. Finally, those with greater future orientation were less likely to fall under the Retreater, Continuer or Searcher view but were more likely to view retirement as a New Beginning.

Regarding the relationship between counterculture and retirement view, the results of Model 2 echo those observed in Model 1, with counterculture remaining a statistically significant predictor of retirement views despite controlling for potential confounders. The more participants identified with counterculture, the more likely they were to identify with either the New Beginning, Continuer and to a lesser extent the Searcher retirement view when compared to the baseline category Freedom From Work.

**Figure 2.**
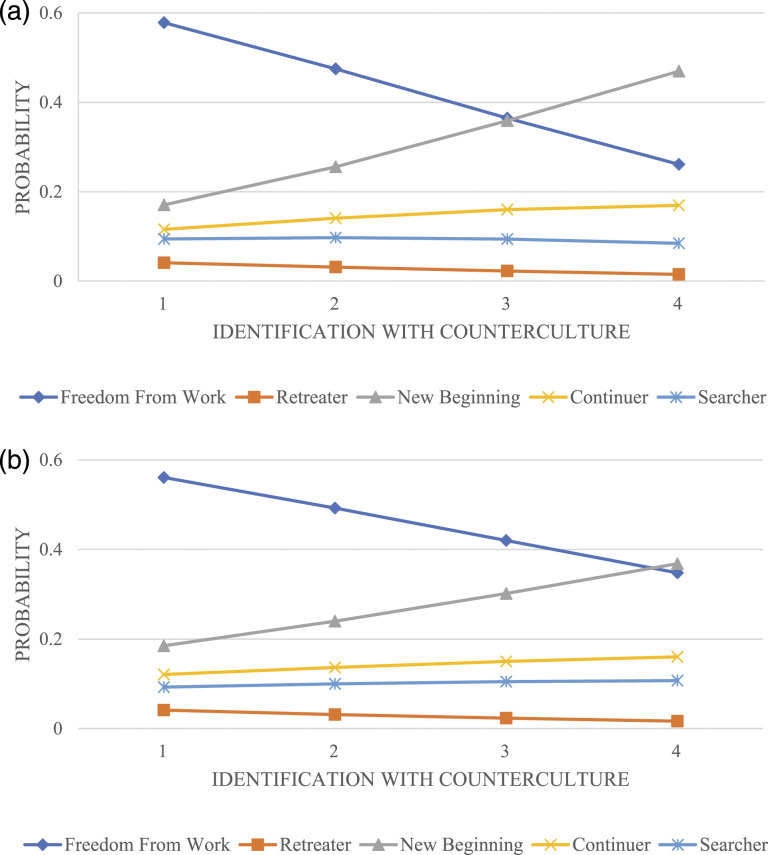
(a) Predictive margins for identification with retirement view based on counterculture, *without* the inclusion of control variables. (b) Predictive margins for identification with retirement view based on counterculture, *with* the inclusion of control variables.

To further illustrate the relationship between counterculture and retirement view, we computed the predicted values of participants’ likelihood to select each of the five retirement views across levels countercultural identification both without (Figure 2(a)) and with (Figure 2(b)) the inclusion of control variables. Figure 2(a) shows that those with greater identification with counterculture are much less likely to view retirement as a life phase of Freedom From Work. While those with the lowest scores on counterculture variable have a 60% likelihood of seeing retirement as a phase of rest and relief from work, this percentage is much lower (30%) among those with a strong identification with the counterculture. The opposite pattern is observed for the category New Beginning, with the probability of identification with this retirement view increasing sharply with greater levels of countercultural identification. Figure 2(b) illustrates that a similar, albeit less pronounced, pattern of association between counterculture and retirement view is observed with the inclusion of control variables.

## Discussion

The retirement of the baby boom generation has prompted much speculation about possible changes they may make to life’s third age, including the notion that the cultural revolution they witnessed in youth has been instrumental in shaping their life course (Gilleard & Higgs, 2002), and retirement views (Hamilton & Hamilton, 2006). In line with our hypotheses, our results support the idea that countercultural identification is associated with retirement views. Those who identified more with countercultural movements in their youth were more likely to identify with active retirement views such as the new beginning and continuer retirement views. Similarly, countercultural identification was negatively associated with the more inactive, traditional view of retirement as a time to enjoy no longer working. From a theoretical perspective, our results support not only the specific idea of the importance of the culture of the sixties and seventies in the retirement views of boomers (Gilleard & Higgs, 2002), but more broadly, the importance of identity formed in youth and its enduring impact on the life course (Kehily, 2007; Kinney, 1993).

That those who identified more with counterculture in their youth are more likely to envision a more active retirement could indicate that those who were at the forefront of countercultural movements in the sixties and seventies may be trailblazers to this day; and may be forerunners of the transformation of retirement expected from the baby boom cohort. A key question emerging from this finding is how enduring are potential changes to retirement brought about or sustained by members of this generation likely to be? If identification with countercultural movements of their youth is associated with baby boomers’ retirement views, should we expect their views, and any subsequent changes to retirement, to endure in succeeding generations not exposed to the same cultural climate? We posit, in line with second demographic transition theory (Van de Kaa, 1987) and diffusion of innovation theory (Rogers & Shoemaker, 1971), that those who identified strongly with the countercultures of the sixties and seventies represent early adopters of lasting social change which is likely to continue to ripple outward. While it is beyond the scope of the current work to ascertain whether changes baby boomers may make to retirement truly represent innovations or are adopted, at least in part, from their predecessors (Chambré & Netting, 2018), we believe that any such changes may likely mark a cultural shift in the field of retirement.

Countercultural identification may also be associated with other domains of older adulthood in which values play a role. It may therefore be interesting to examine baby boomers’ attitudes towards topics such as old age more generally, assisted living and other long-term supports and services (Robison, Shugrue, Fortinsky, & Gruman, 2013), and attitudes towards death and dying (Yi & Hong, 2016) through the lens of youth (counter)cultural identity. Some additional insights emerging from our study relate to other factors that may be associated with retirement views. Although included primarily as a control, our results indicate that education may be a strong predictor of some retirement views, with better educated individuals more likely to view retirement as a time for self-development or new beginning or a time to continue their work at a slower, more self-directed, pace. These findings are in line with previous works finding a strong educational gradient in views on retirement (Henkens & van Solinge, 2021). Given that educational level is likely to be higher in future cohorts the trend towards retirement as a time of learning and self-development is likely to continue to grow.

Our study has several strengths. First, to the best of our knowledge, this is the first large-scale quantitative study exploring the long-term impact of identification with countercultures, on retirement. Second, not only does our study investigate the previously understudied retirement views of baby boomers, but we examine these through a cultural lens rather than the traditional cohort approach. We believe this novel cultural approach offers a more well-rounded understanding of retirement views as well as capturing the diversity of this cohort; both in terms of retirement views and identification with counterculture, and along demographic and social lines.

While our study makes several contributions to the literature, it is not without limitations. No information regarding other potentially relevant psychological predictors of retirement views, such as personality factors was available. Furthermore, our study investigates the impact of countercultural identity on retirement views in one specific country context. Given disparities between retirement systems and pension schemes – and the heterogeneity in countercultural movements worldwide (Marwick, 2011), it is plausible that findings may vary in other countries with a less generous retirement system, or that underwent differing levels of societal and cultural change in the sixties and seventies. Additionally, our independent variable is retrospective in nature. The broad scope of this variable – measuring identification with and connection to particular cultural movements – means it is likely to rely on semantic aspects of autobiographical memory. Therefore, it may be less vulnerable to inaccuracies than were we to investigate more specific, episodic, aspects of autobiographical memory (St Jacques & Levine, 2007; Tulving, 2002), it is nevertheless possible that the retrospective nature of this measure may have some impact on its accuracy. Finally, the data in this study is cross-sectional, limiting the causal inferences that can be drawn from the results.

The prospect of baby boomers reaching old age has often been met with trepidation by researchers, clinicians and policy makers alike (Knickman & Snell, 2002). However, understanding the underlying views and values of this generation may be crucial in understanding their transition to later life and how successful this will be (Huber & Skidmore, 2003). To this end, our study has provided important initial insights into how baby boomers view their retirement, and possible links between these views and their unique cultural upbringing during the sixties and seventies. What remains to be seen however, is whether, and to what extent, the baby boom generation will change the nature of retirement as an institution, and what repercussions these changes may have for generations to come.

### Note

To gain additional insight into the relationship between countercultural identification and education, we examined the correlation between the individual items making up our countercultural measure and education level. The following correlation coefficients were reported between Educational level and individual elements of our countercultural measure: hippy movement (*r* = .10), protest generation (*r* = .29), individualism (*r* = .14), feminism (*r* = .26), drugs culture (*r* = .01), anti-establishment (*r* = .25) and alternative lifestyles (*r* = .20).

## Appendix

**Table:** Results of Separate Logistic Regression Analyses Investigating Association Between Countercultural Identification and Retirement Views.

Variable	Freedom From Work Coef. *(SE)*	Retreater *Coef.(SE)*	New Beginning *Coef. (SE)*	Continuer *Coef. (SE)*	Searcher *Coef. (SE)*
*Model 1: Key predictor variable only*					
Counterculture	−0.12 (.05)*	−0.18 (.11)	0.37 (.05)***	0.16 (.06)*	−0.12 (.08)
*Model 2: Controls included*					
Counterculture	−0.09 (.06)	−0.08 (.11)	0.34 (.06)***	0.18 (.07)**	0.02 (.08)
*N*	5942	5825	5876	5844	5832
Identification with retirement view	55.97%	7.74%	38.51%	19.13%	15.48%

*Note.* Model 2 includes control variables used in the primary analysis, but their coefficients are omitted here for the sake of brevity.
